# Supplementary material for: Usp22 Deficiency Leads to Downregulation of PD-L1 and Pathological Activation of CD8+ T Cells and Causes Immunopathology in Response to Acute LCMV Infection
Source: Vaccines (Basel). 2023 Oct 5;11(10):1563. doi: 10.3390/vaccines11101563 (PMC10610587; doi:10.3390/vaccines11101563)
Supplement: Supplementary file 1 [file vaccines-11-01563-s001.zip › vaccines-2547387-supplementary.pdf]

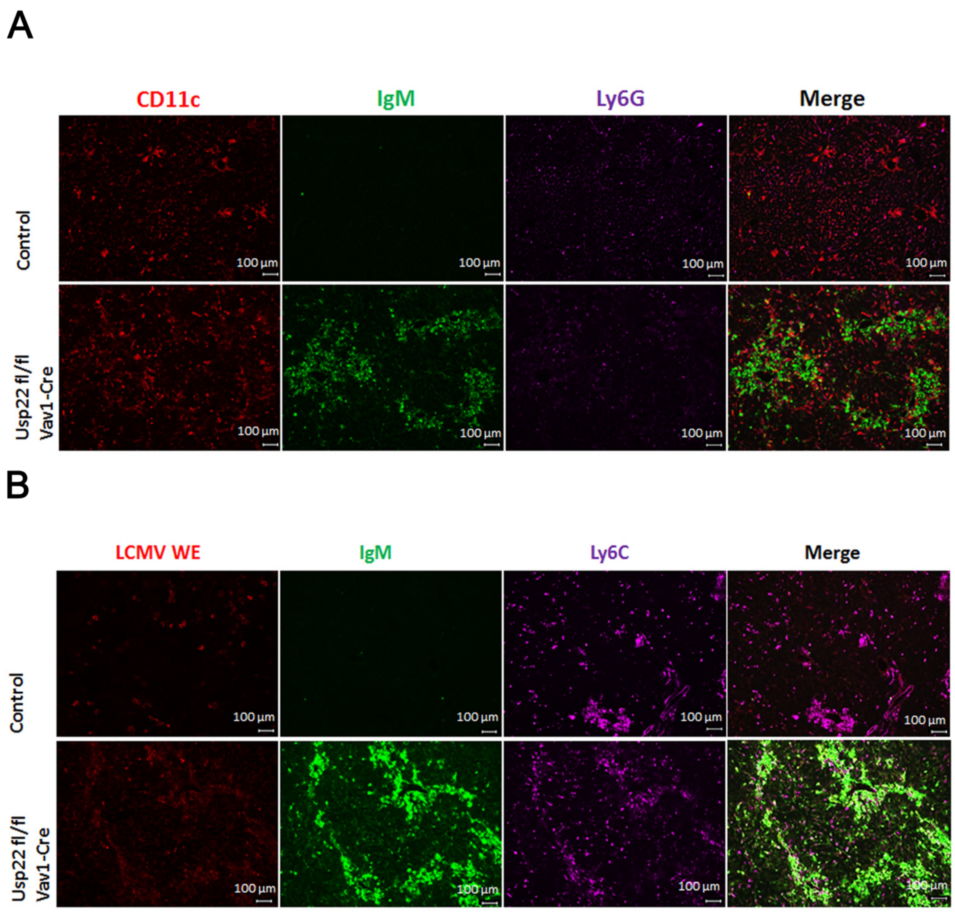

Supplementary Figure S1. Colocalization of infiltrates containing neutrophil granulocytes and monocytes with IgM deposits (A) and LCMV virus particles (B). (A-B) 2× 10^5 PFU of the LCMV WE were administrated intravenously per mouse on day 0. Immunofluorescence analysis of liver sections collected on day 9 after infection shows LCMV nucleoprotein, IgM and CD11c and Ly6G positive cells. Scale bar = 100 µm; one representative out of 6 is shown. Fluorescent microscopy images were captured at 10x magnification using Keyence BZ-9000E microscope.

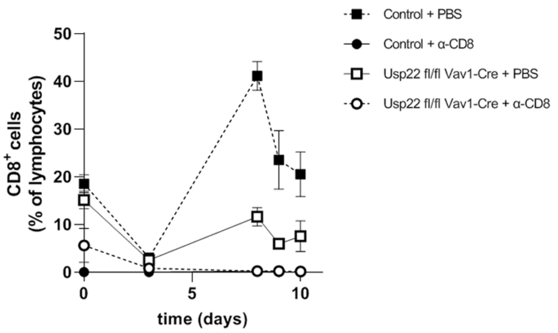

Supplementary Figure S2. Sufficient depletion of CD8⁺ T cells under treatment with the monoclonal anti-CD8a antibody. Prior to the LCMV WE infection (2× 10^5 PFU per mouse), Usp22 deficient mice and WT were treated intraperitoneally with 100 ug of the monoclo-nal anti-CD8a antibody per mouse on day -1, 0 and then every second day. PBS served as control. Intravenous infection with LCMV WE (2× 10^5 PFU per mouse) was done on day 0. Numbers of $CD8^+$ T cells were determined by flow cytometry in peripheral blood samples obtained at indicted time points after infection (n = 6). Data from two independent experiments with consistent results are shown.

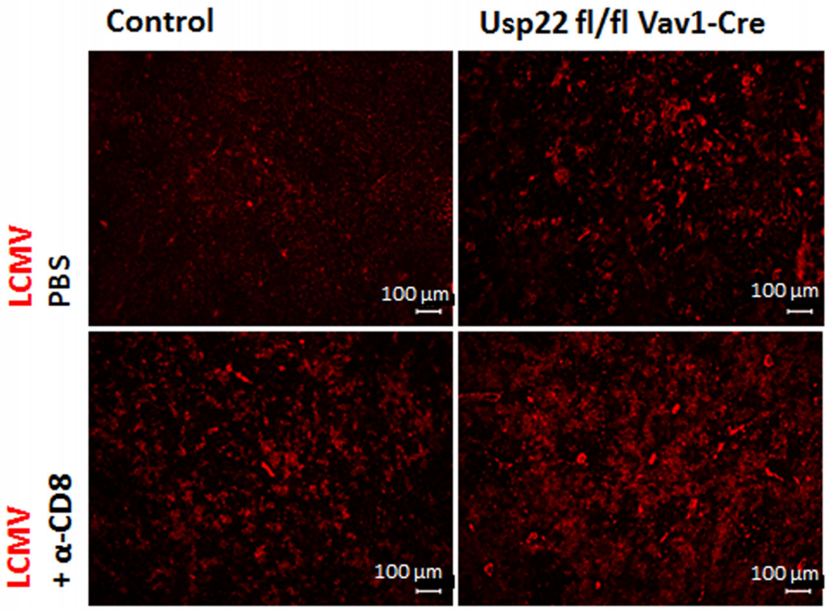

Supplementary Figure S3. Depletion of $CD8^+$ T cells results in increased LCMV load in Usp22 deficient mice. Before infection with LCMV WE (2× 10^5 PFU per mouse), Usp22 deficient mice and WT mice were treated intraperitoneally with 100 µg of the monoclonal anti-CD8a antibody per mouse on day -1, day 0, and then every second day. PBS served as a control. Intravenous infection with LCMV WE (2× 10^5 PFU per mouse) was per-formed on day 0. Immunofluorescence staining of snap-frozen liver sections was per-formed on day 9 after depletion of $CD8^+$ T cells with subsequent infection. Scale bar = 100 µm; one representative out of 6 is shown. Fluorescence microscopy images were captured at 10×x magnification with a using Keyence BZ-9000E microscope.
